# Molecular Dynamics Simulation on the Diffusion of Flavor, O_2_ and H_2_O Molecules in LDPE Film

**DOI:** 10.3390/ma12213515

**Published:** 2019-10-26

**Authors:** Binqing Sun, Lixin Lu, Yong Zhu

**Affiliations:** 1 Department of Packaging Engineering, Jiangnan University, Wuxi 214122, China; sunbinqing@tust.edu.cn; 2 Department of Packaging Engineering, Tianjin University of Science and Technology, Tianjin 300222, China; 3 Department of Packaging Engineering, Jinan University, Zhuhai 519070, China; zy792@139.com

**Keywords:** flavor organic molecules, LDPE film, molecular dynamics simulation, diffusion

## Abstract

The diffusion of five flavor organic molecules, including D-limonene, myrcene, ethyl hexanoate, 2-nonanone, and linalool in low density polyethylene (LDPE) film were investigated by combined experimental and molecular dynamics (MD) simulation studies. The diffusion coefficients derived from the prediction model, experimental determination, and MD simulation were compared, and the related microscopic diffusion mechanism was investigated. The effects of the presence of the flavor organic molecules on the diffusion of O_2_ and H_2_O in polyethylene (PE) were also studied by MD simulation. Results show that: The diffusion of five flavor molecules in LDPE is well followed to Fick’s second law by the immersion experiment; MD simulation indicates the dual mode diffusion mechanism of the flavor molecules is in LDPE; the diffusion coefficients from MD simulation are close to those from the experimental determination, but are slightly larger than those values; the presence of the flavor organic molecules hinders the diffusion of O_2_ and H_2_O, which can be well explained from the fraction of free volume (*FFV*) and interaction energy calculation results derived from MD simulation.

## 1. Introduction

Polyethylene (PE) is a kind of thermoplastic, which has excellent chemical stability, heat sealing, toughness and high elasticity, as well as cold resistance and water vapor barrier [1,2]. Low density polyethylene (LDPE) is commonly used in food packaging because of its excellent thermal sealing and chemical stability [3]. Food flavor is an important quality characteristic of food [4]. It is reflected by volatile compounds in food, which determines the quality of food [5,6,7]. Food flavor molecules will be adsorbed by packaging materials after long-term storage, and the change of its quantity will lead to the change of food flavor, thus, affecting food sales and consumer complaints [8].

The adsorption of food flavor molecules in packaging materials mainly depends on the characteristics of flavor molecules, packaging materials, storage environment and other factors, which subsequently changes the flavor of food or the properties of polymer materials [9]. During the last decades, many researchers have studied the adsorption of flavor molecules into different packaging materials, such as PE [3,10,11,12,13], polypropylene (PP) [14], polyethylene teraphthalate (PET) [5,15], polylactate (PLA) [13], etc. In packaging materials, the flavor adsorption amount of the rubber polymer (e.g., PE) is several thousand times higher than that of the glass polymer, such as PET [16]. The adsorbable flavor organic molecules of LDPE film have been extensively studied, including the alkenes, the esters, the aldehydes, the ketones, and the alcohols [3,10,11,12,13,17,18]. The flavor of liquid food is easier to be lost than that of solid food [19]. The concentration range of flavor organic molecules in liquid food is ppm or less, which is within the threshold range of human taste [20].

The molecular dynamics simulation method is a good tool to illustrate the microscopic phenomenon of the molecule mass transfer in the packaging materials [21]. More detailed information and basic principles of the interaction between molecules and polymers can be obtained [22,23,24,25]. Börjesson et al. performed Monte Carlo (MC) and MD simulation to calculate solubility coefficients and diffusion coefficients of oxygen and water in PE to obtain a microscopic diffusion mechanism [26]. Yue et al. used MD method to study the diffusion of benzene, toluene and ethyl benzene in amorphous PE membrane, and found the simulation results had the same trend as the experimental results [27]. Wang et al. determined the diffusion coefficients of molecules with different molecular weights in amorphous PET by MD simulation [28]. Tao et al. used Grand canonical Monte Carlo (GCMC) and MD simulation to study the adsorption and diffusion of O_2_ in PP and showed that the loading of O_2_ increased and its diffusion coefficient in PP decreased with the increase in the polymerization degree of PP [29]. However, the adsorption of food flavor molecules in LDPE film was mainly studied by experiment [3,4,5,7], and there were few studies on the adsorption and diffusion of flavor substances in LDPE by MD simulation. It is also meaningful to analyze the diffusion behavior of flavor organic molecules in PE and their effects on the diffusion of O_2_ and H_2_O in LDPE from the microscopic view.

In this work, the diffusion coefficients of food flavor organic molecules in LDPE film were studied by two-sided immersion experiment, Brandsch prediction model and MD simulation. The differences in diffusion coefficients obtained by the above three methods were compared. By calculating the interaction energies between the molecules and PE chain, the interaction energies between O_2_ or H_2_O molecule and flavor molecules, and the *FFV* of PE cell, the related diffusion mechanism of diffusion molecules in LDPE film was further analyzed. Furthermore, the diffusion coefficients of O_2_ and H_2_O in LDPE film before and after adsorption were calculated by the MD method, and the effects of flavor molecules on the diffusion of O_2_ and H_2_O were investigated.

## 2. Materials and Methods

### 2.1. Materials

LDPE film with a thickness of 47.76 μm, and a density of 0.923 g/cm^3^ was donated by Tianjin Baihao Co., Ltd. (Tianjin, China). The chemical reagents used and their corresponding purity, and CAS numbers were as follows: D-limonene (D-LE, 99%, 5989-27-5) from Adamas (Innochem, Beijing China); myrcene (ME, 99%, 123-35-3) from Aladdin (Shanghai Aladdin Biochem. Tech., Shanghai, China); ethyl hexanoate (EH, 99%, 123-66-0) from TCI (J&K Chemical, Beijing, China); 2-nonanone (2-NO, 99%, 821-55-6), and linalool (LO, 98%, 78-70-6) from Macklin (Tianjin Sanjiang, Tianjin, China); anhydrous ethanol (99%, 64-17-5) and n-hexane (99%, 110-54-3) from Jiangtian (Tianjin Jiangtian, Tianjin, China). D-limonene, myrcene, ethyl hexanoate, 2-nonanone, and linalool were selected as volatile flavor molecules in liquid foods, such as fruit juice and liquor [30,31], and 10% (*V*/*V*) aqueous ethanol solution was selected as the food simulation solution.

### 2.2. Film Adsorption Experiment

The contact process between liquid food and LDPE film was simulated by two-sided immersion method in the food simulation solution [10]. Five flavor reagents were mixed together into 10% (*V*/*V*) aqueous ethanol solution with a concentration of 500 mg/L for each flavor reagent to prepare a liquid simulator [32]. According to the contact ratio of actual food to material, a 5 cm × 3 cm film was placed into a 20 mL glass bottle with 20 mL of food simulator [33]. The bottle cap was filled with the polytetrafluoroethylene (PTFE) gasket. After the cap was sealed, the outer part was wound with a sealing film to ensure sealability. Storage temperature was set as 23 °C. After storage for a certain period of time, the film was taken out and repeatedly wiped with test paper until dry. The film was then cut into small pieces and placed in a 20 mL n-hexane sample bottle, and then sealed with the PTFE gasket and sealing film. Then the film was extracted in the ultrasonic water bath pot for 1 h. The ultrasonic wave frequency was 60 Hz, and the temperature was 23 °C. Sample bottles after extraction were stored at room temperature for 24 h. The amount of extraction sample was measured by Chromatography-Mass Spectrometer (GC-MS).

### 2.3. GC-MS Analysis Conditions

GCMS-QP2010 (Shimadzu, Kyoto, Japan) was used to measure the adsorption amount of flavor. The external standard method was used to analyze the measured flavor quantitatively. The GC-MS analysis conditions were as follows: The chromatographic column was DB-5 quartz capillary column (30 m × 0.25 mm × 0.25 µm), the detector was hydrogen flame ionization detector, and the injection volume was 1 µL. The inlet temperature was 250 °C, with a shunt ratio of 20:1, and the ion source temperature was 200 °C. The column temperature was programmed to an initial temperature of 40 °C, which was maintained for 3 min. Column was heated up to 150 °C at the rate of 4 °C/min, and then maintained at 150 °C for 1 min. Then it was heated up to 250 °C at a rate of 8 °C/min and maintained at 250 °C for 6 min.

### 2.4. Data Analysis

In order to better analyze the amount of flavor adsorbed by packaging film, the data measured by GC-MS need to be standardized. All the data were converted by Equation (1) to obtain the adsorption amount per unit mass of packaging film [3,10]:

(1) $$M_{i,t}=\frac{C_{i}\times \rho_{i}\times V_{E}}{M_{P}},$$

where *M_i,t_* is the adsorption amount of flavor *i* per unit mass of packaging film at time *t*, mg/g; *C_i_* is the concentration (mL/mL) of flavor *i* in the extract solution (n-hexane) detected by GC-MS; *V*_E_ is the volume of the extract solution (n-hexane), mL; *ρ_i_* is the density (mg/mL) of flavor *i* and *M*_P_ (g) is the quality of the adsorption packaging film.

## 3. Molecular Simulation Details

### 3.1. Modeling

**Figure 1:**
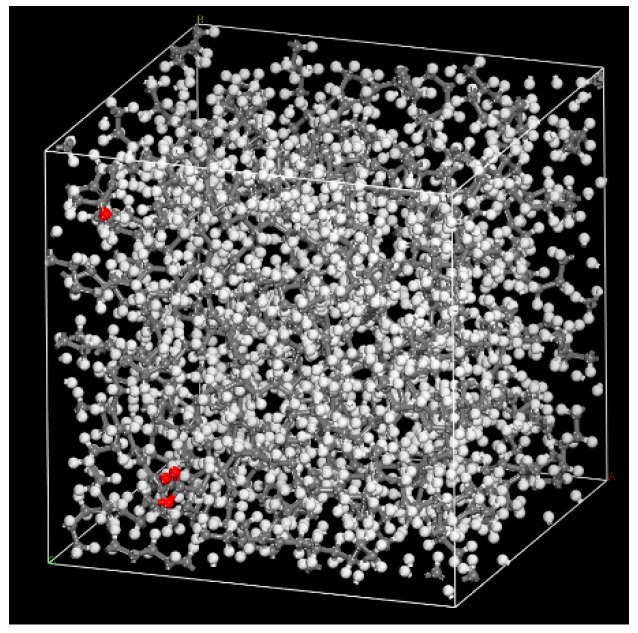
Amorphous cell of polyethylene (PE) and five flavor organic molecules.

The molecular models of five flavor organic molecules and PE were constructed with the Materials Visualizer module of Materials Studio 8.0 (MS 8.0). Generally, setting a higher polymerization degree for PE can simulate a more realistic diffusion of molecules in PE [27]. However, considering the computational complexity, a polymerization degree of 160 was selected to build the PE chain (PE 160). After the competition of modeling, the above models were geometrically optimized by using Forcite module with the “Smart Minimizer” method. The amorphous cells containing the molecules and PE chains were built to simulate the composition of the sample prepared in this study (as listed in Table 1). In order to analyze the diffusion of O_2_ and H_2_O in LDPE films, three O_2_ or ten H_2_O were placed into the cell structure of PE 160. The simulation cells with the initial density of 0.923 g/cm^3^ were constructed according to the actual density of LDPE film. Figure 1 shows an example of the simulation cells.

### 3.2. Simulations

**Figure 2:**
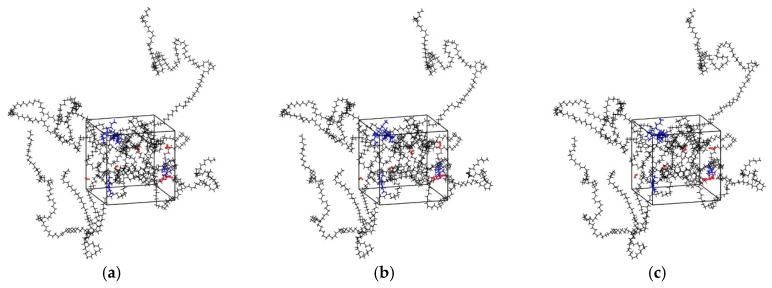
Snapshots of the simulation boxes at different simulation time (black: PE; red: H_2_O molecules; blue: Flavor molecules). (**a**) t = 0; (**b**) t = 50 ns; (**c**) t = 100 ns.

The main procedures of simulation included geometry optimization, annealing and dynamic simulations, using the COMPASS II force field in all stages which is a newer version of the COMPASS force field and mainly used to simulate polymers, metals, metal oxides and small molecules [34]. The Coulomb force was evaluated by the Ewald summation method with an accuracy of 10^−3^ kcal/mol, and the van der Waals force was computed by the atom-based summation method with a spline cutoff distance of 12.5 Å. The geometric optimization procedure was used to minimize the energy of the cell. The isothermal-isobaric ensemble (NPT) was selected to perform in the molecular dynamics simulations. Compared with other ensembles, NPT simulation is the most relaxed ensemble in the system [35]. The pressure of annealing simulation was set as 0.1 MPa controlled by Berendsen method. The initial temperature was 300 K, and the mid-cycle temperature was 600 K. Three annealing cycles were performed, and the total number of steps was 3.6 × 10^5^. After the annealing, the internal stress of cell decreased, and the unreasonable structure during cell construction was eliminated [36]. Then, the NPT MD simulation was performed for 1000 ps to reach balance and the other constant volume and temperature ensemble (NVT) MD simulation of 100 ns was performed to ensure the convergence of the MD simulation for diffusion analysis. It was found that the diffusion coefficients calculated by allowing only the diffusing species to move, but keeping fixed the rest of the system can be affected by significant errors [37,38]. Therefore, during all the simulations in this study, all the atoms, including the atoms of five flavor molecules, O_2_, H_2_O, and LDPE, were allowed to move (see Figure 2).

## 4. Results and discussions

### 4.1. Interaction Energy between Diffusion Molecules and PE Chain

The interaction energy (*E*_int_), indicating the intensity of interaction between the diffusion molecule and polymer, is derived according to the following equation:

(2) $$E_{\mathrm{int}}=E_{\mathrm{total}}-E_{\mathrm{PE}}-E_{\mathrm{diff},i}$$

where *E*_total_ is the total energy of the system, *E*_PE_ is the energy of PE chain, *E*_diff,*i*_ is the energy of diffusion molecule *i* in the PE chain. A negative *E*_int_ value is corresponding to stable interaction between the components [39,40]. More negative *E*_int_ indicates a stronger interaction in the system.

In this study, there was only physical bonding between diffusion molecules and polymer chains, and no chemical reaction and new chemical bonds involved. The non-bonding interactions between diffusion molecules and polymer chains, and the interatomic potential for the interactions among LDPE atoms and diffusion molecules were considered. The interaction energies of D-limonene, myrcene, ethyl hexanoate, 2-nonanone, linalool, O_2_ and H_2_O with PE chain were calculated by MD simulation and were listed in Table 2. The stronger the interaction between diffusion molecules and polymer chains will result in the higher the energy barrier to be overcome and the smaller the corresponding diffusion coefficient. Therefore, if only take the effect of interaction energy on the diffusion into account, the diffusion coefficients of the diffusion molecules in PE should decrease in the following order: *D*_H2O_ > *D*_O2_ > *D*_D-__LE_ > *D*_2-__NO_ > *D*_ME_ > *D*_EH_ > *D*_LO_. However, besides the interaction energy, molecular size and free volume of polymer cell are also important factors affecting the diffusion of molecules, which will be further discussed in a later section.

### 4.2. Diffusion Coefficient (D)

#### 4.2.1. Calculation of D by Prediction Model

Diffusion coefficient is the most important analytical parameter in adsorption of flavor organic molecules by polymers. The classical prediction model of diffusion coefficient, Brandsch model (Equation (3)), was widely used to predict the *D* of molecules in polymers [41].

Brandsch model introduced two simplification factors, *A*_P_ and *τ*, to describe the properties of polymer. Brandsch model was recognized as a general formula for characterizing the diffusion coefficient of molecule in plastic packaging film [42]:

(3) $$D=10^{4}\exp\left(A_{P}-0.1351M_{r}^{2/3}+0.003M_{r}-\frac{10454}{T}\right),$$

where *D* is the diffusion coefficient of the diffusion molecule in polymer (cm^2^/s), *M*_r_ is the relative molecular weight of the diffusion molecule, *A*_P_ is the characteristic parameter of polymer, and *T* is the absolute temperature (K). For different polymer matrix, the value of *A*_P_ is different, which is mainly derived from a large number of experimental results. *A*_P_ is usually a function of temperature, and it can be calculated from the below equation:

(4) $$A_{P}=A_{P}^{*}-\tau /T,$$

where *A*_P_^*^ is the adiabatic term of polymer, and *τ* is the deviation of diffusion activation energy from reference activation energy in polymer. For LDPE films, *A*_P_^*^ equals 11.5, and *τ* equals 0 [43].

The diffusion coefficients (*D*_pre_) of D-limonene, myrcene, ethyl hexanoate, 2-nonanone and linalool in LDPE were calculated by Equations (3) and (4), as shown in Table 3.

#### 4.2.2. Experimental Determination of D

**Figure 3:**
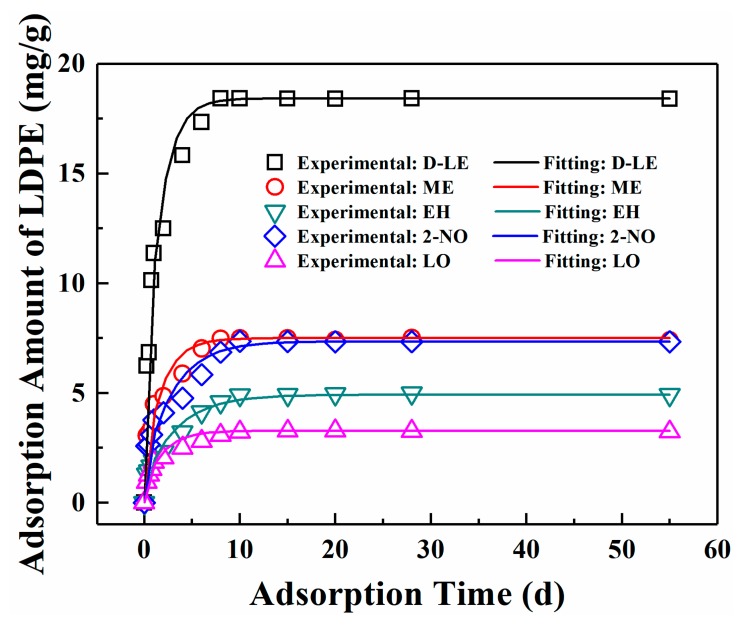
Comparison of experimental data and fitting values.

The adsorption of five flavor organic molecules in LDPE film at 23 °C was studied by film immersion experiment. As shown in Figure 3, for all five flavor organic molecules, adsorption equilibrium can be reached within 10 days. After reaching the adsorption equilibrium, the total amount of D-limonene adsorbed is the largest, i.e., 18.4212 mg/g, followed by myrcene, which is 7.5019 mg/g. For linalool, it has the lowest adsorption amount (3.2382 mg/g) among five flavor organic molecules. The adsorption equilibrium amounts of the organic molecules by LDPE films follow the order: D-limonene > myrcene > 2-nonanone > ethyl hexanoate > linalool. Although molecular weights of D-limonene and myrcene are the same, the adsorption amounts of the two molecules are quite different.

When the adsorption amount of the flavor organic molecule was known, the Fick’s second law diffusion model can be used to calculate the diffusion coefficient [44,45]:

(5) $$\frac{M_{i,t}}{M_{i,e}}=1-\sum_{n=0}^{\infty }\frac{8}{{\left(2n+1\right)}^{2}\pi^{2}}\exp\left[\frac{-D{\left(2n+1\right)}^{2}\pi^{2}t}{4L_{P}^{2}}\right],$$

where *M_i,t_* is the adsorption amount of flavor *i* adsorbed by films at *t* (s) time, mg/g; *M*_*i*,e_ is the adsorption amount of flavor *i* at equilibrium time, mg/g; and *L*_P_ is the thickness of films, cm. The experimental diffusion coefficient, *D* can be derived by fitting the Equation (5) with least square method.

The diffusion coefficients of five flavor organic molecules in LDPE were fitted with the experimental data by using MATLAB Software and listed in Table 4. The comparison between the fitted curve and the experimental scatter points is shown in Figure 3. Table 4 and Figure 3 show that the diffusion of five flavor organic molecules in LDPE well conforms to Fick’s second law.

#### 4.2.3. Molecular Simulation Results

The diffusion coefficient in MD simulation was calculated by analyzing the mean square displacement (MSD) curve. Four methods can be used to analyze the MSD curve, i.e., Einstein method, Green-Kubo method, differential-zone variational method, and cluster analysis method [46].

Einstein method is a classical method, which correlates diffusion coefficient *D* with MSD of molecules [42]. The formula is as follows:

(6) $$D=\frac{1}{6N}\underset{t\to \infty }{\lim}\frac{d}{dt}\sum_{i=1}^{N}\langle {\left|r_{i}\left(t\right)-r_{i}\left(0\right)\right|}^{2}\rangle ,$$

where *N* is the number of diffusion molecule *i* in the system, *t* is the diffusion time, and *r_i_*(0) and *r_i_*(*t*) are the diffusion position vector of diffusion molecule *i* at the time of 0 and *t*, respectively.

**Figure 4:**
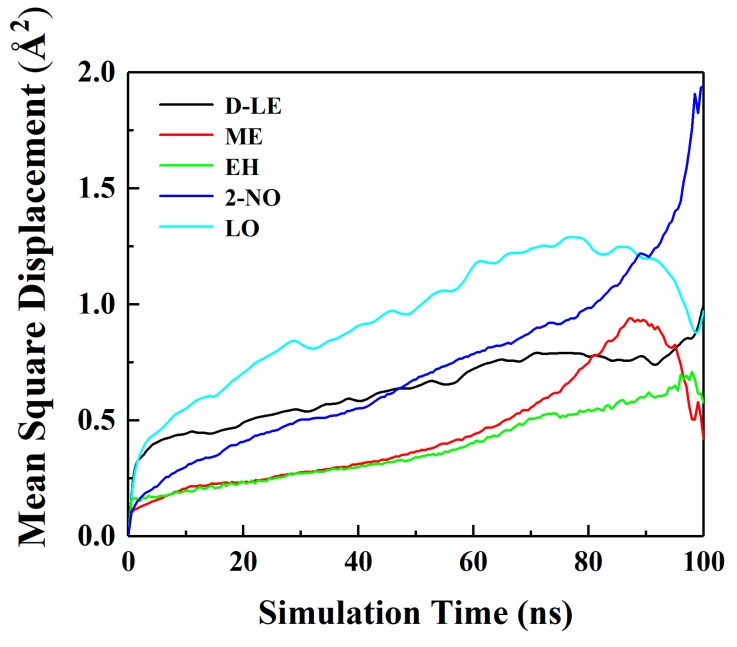
Mean square displacement (MSD) curves of five flavor organic molecules in PE.

**Figure 5:**
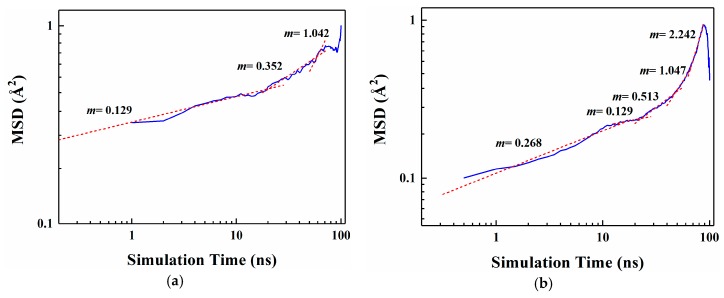

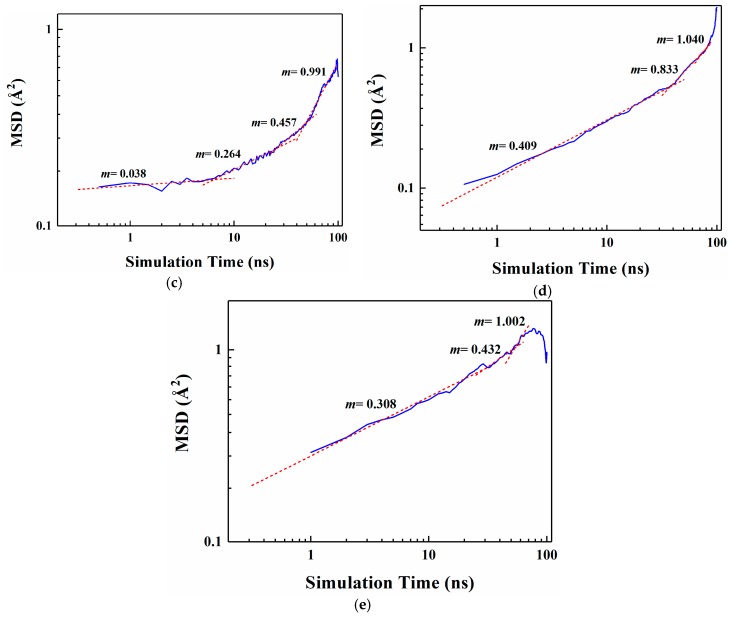
Segmental linear fitting diagrams of *Log*10 curves for five organic molecules MSD. (**a**) D-limonene; (**b**) Myrcene; (**c**) Ethyl hexanoate; (**d**) 2-nonanone; (**e**) Linalool.

Figure 4 shows the MSD curves of five flavor organic molecules in PE derived from MD simulation. All MSD curves contain the linear part, unstable vibration part and the noisy part after a long simulation time. The non-linear and noisy parts usually encountered at the end of the curves may be attributed to the statistical error after longer simulation times. Reynier proposed that there were three diffusion modes of molecules in polymers: Crawling diffusion mode, jumping diffusion mode, and dual mode diffusion [47,48]. The flavor molecules (D-limonene, myrcene, ethyl hexanoate, 2-nonanone and linalool) contain both rigid and flexible groups; therefore, their diffusion may follow the dual mode diffusion (both the jumping and crawling and diffuse modes involved). Figure 4 shows that flavor organic molecules, because of their large size, cannot jump easily and diffuse in polymers like small gas molecules, and the linear part and unstable vibration part of their MSD curves show dual mode diffusion. In order to analyze molecular diffusion behavior more clearly, the MSD curves of five organic molecules are calculated by *Log*10 and fitted by segments (as shown in Figure 5). In Figure 5, *m* is the slope of the fitting straight line, which is anomalous (non-Einstein) behavior when *m* < 1, and Einstein behavior when *m* is close to 1 [49]. As can be seen from the figure, at the back end of the simulation time, five organic molecules transform from anomalous diffusion to Einstein diffusion. During this time, the diffusion coefficient can be calculated by Einstein Equation (6). The diffusion coefficients of five flavor molecules (D-limonene, myrcene, ethyl hexanoate, 2-nonanone and linalool) in PE are 9.31 × 10^−11^ cm^2^/s, 7.90 × 10^−11^ cm^2^/s, 6.90 × 10^−11^ cm^2^/s, 1.56 × 10^−10^ cm^2^/s, 1.72 × 10^−10^ cm^2^/s, respectively.

#### 4.2.4. Comparison among Experiment, Prediction Model and MD Simulation Results

The differences among prediction model diffusion coefficient (*D*_pre_), experimental diffusion coefficient (*D*_exp_) and simulated diffusion coefficient (*D*_sim_) were compared as listed in Table 5. The *D*_pre_ is higher than that obtained by experiments and MD simulation, which is similar to the results reported by Wang et al. [50] who investigated the diffusion of small molecules in PET. This is because Brandsch prediction model is based on the assumptions given under deteriorating conditions, and it only considers the properties of polymer substrates, the molecular weight of flavor molecules and the temperature, but does not take the interaction between molecules into account.

The diffusion coefficients obtained by MD simulation are close to those of experimental results, but are slightly larger than those values. This difference may be due to: (1) the setting of finite chain length for PE means that many relaxed free ends make the redistribution of free volume fraction easier and lead to the increase of diffusion coefficient. The simulation results reported by Wang et al. [50] showed a good agreement with that obtained from experiments, this may be due to the PET models built in their study had a polymerization degree of 100, which is close to that of actual PET. Typically, the actual LDPE has a polymerization degree of >1000. Building an LDPE model with such a high polymerization degree will lead to a very large model and an unbearable expense of calculation time in the subsequent MD simulation. (2) The amorphous PE cell was constructed for MD simulation, but the actual PE is an incomplete amorphous polymer which contains a certain crystalline phase. The crystallization zone will hinder the diffusion of flavor organic molecules. (3) MD simulation only considers the diffusion of flavor organic molecules in PE materials, and does not consider the distribution of flavor organic molecules from food simulation solution to PE material, which will lead to the deviation between MD simulation and experimental values. However, it can be concluded that the simulation results are close to the experimental ones by taking the errors of these numbers (listed in Table 5) into account. In addition, the experimental process was more complex than the predictive model and MD simulation. Because the immersion of food simulation solution, i.e., ethanol and water, may be easier to first diffuse into the material than flavor molecules, thus, maybe hinder the diffusion of flavor molecules.

### 4.3. Fraction of Free Volume

The size and shape of a free volume play an important role in the diffusion behavior of diffusion molecules in polymers. Generally, the larger the free volume fraction of polymers results in the larger the diffusion coefficient. When calculating the free volume of the system, the hard sphere probe model in MS 8.0 was used to analyze the kinetic radius of the diffusion molecule. The free volume and occupied volume of the cell can be obtained when the molecular probe with a certain radius *R*_P_ moves on the van der Waals surface. The ratio of free volume to simulated cell volume is defined as *FFV* [51]:

(7) $$FFV=\frac{V_{F}}{V_{S}}=\frac{V_{F}}{V_{F}+V_{O}},$$

where, *V*_F_ is the free volume of the polymer, *V*_O_ is the occupied volume of the polymer, and *V*_S_ is the total volume of the polymer.

**Figure 6:**
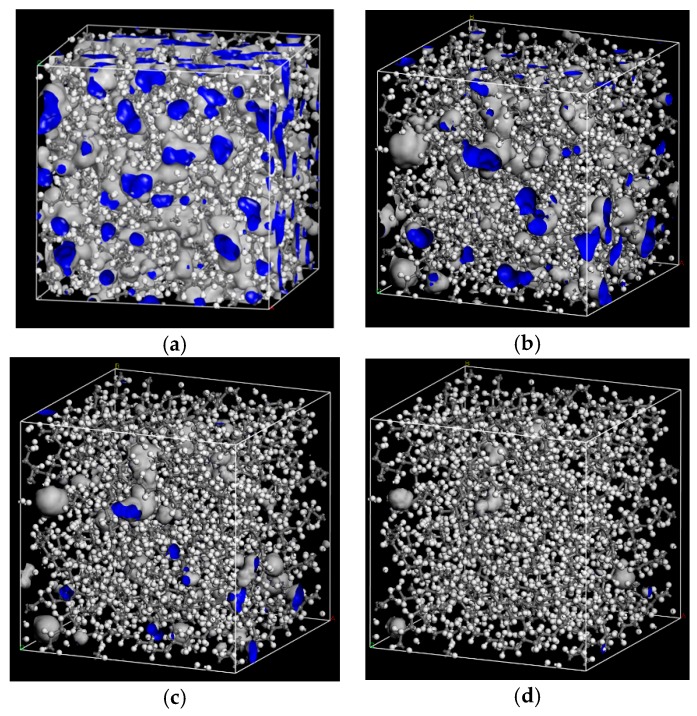
Free volume morphologies of the different probe radius in PE cell. (**a**) *R*_P_ = 1 Å; (**b**) *R*_P_ =1.5 Å; (**c**) *R*_P_ =2 Å; (**d**) *R*_P_ =2.7 Å.

The probe radii of 1.0 Å, 1.5 Å, 2.0 Å, 2.7 Å were selected to calculate the free volume of PE cells. Free volume morphologies with the different probe radius in PE cell are shown in Figure 6.

Table 6 shows that with the increase of *R*_P_, the *FFV* of PE cell decreases. The free volume measured by 2.7 Å can meet the space requirement of jump diffusion for O_2_ and H_2_O. It also means that for larger flavor organic molecules, there are fewer free holes to jump. The polymer chains can wriggle themselves, and when their wriggling creates enough jumping space, flavor organic molecules will jump from one hole to other holes.

### 4.4. Diffusion of O_2_ and H_2_O in PE before and after Adsorption

**Figure 7:**
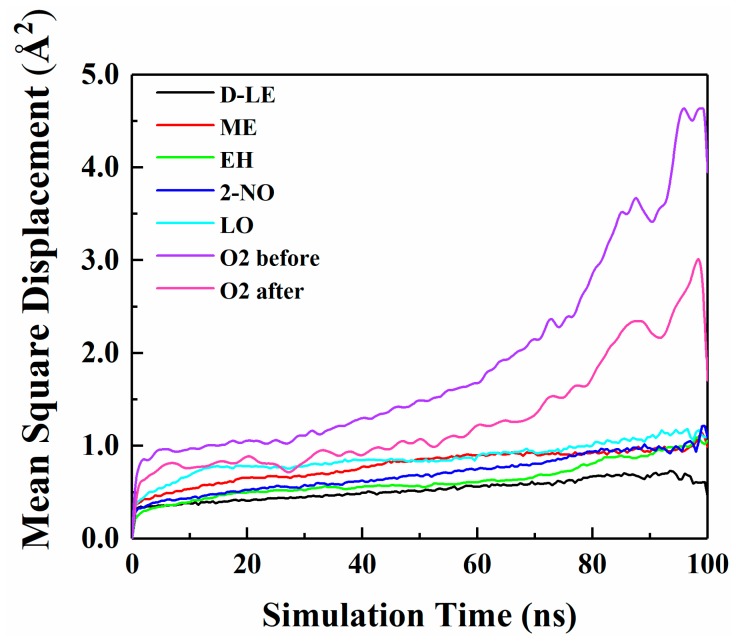
MSD curves of O_2_ and five flavor organic molecules in PE.

**Figure 8:**
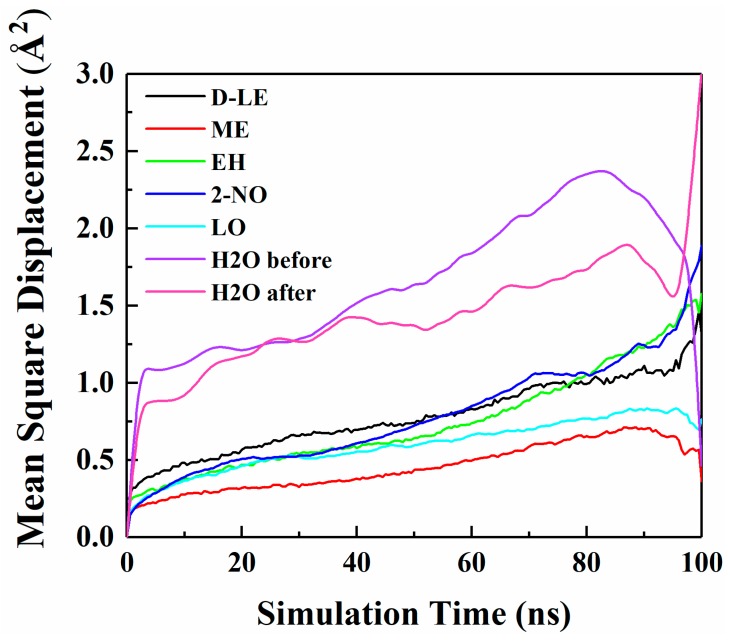
MSD curves of H_2_O and five flavor organic molecules in PE.

The diffusion of O_2_ and H_2_O in PE before and after adsorption of flavor molecules were simulated by the MD method, and the related MSD curves were shown in Figure 7 and Figure 8. In the long-term MD simulation, the diffusion of molecules in random motion is affected by the steric hindrance of PE chain, resulting in a decrease of diffusion coefficient. Compared with Figure 4, O_2_ or H_2_O compete with the flavor molecules for free holes in PE, which restricts the diffusion of O_2_, H_2_O and flavor molecules. After adsorption of flavor molecules, O_2_ and H_2_O diffuse faster than all flavors, and their MSD curves increase more rapidly. The reason is that O_2_ and H_2_O are smaller than flavor molecules and need less diffusion space. Moreover, the effect of flavor molecules on O_2_ is small; therefore, O_2_ diffusion is faster than H_2_O.

The diffusion coefficients of O_2_ and H_2_O before and after PE adsorption are listed in Table 7. Table 7 indicates that the diffusion of O_2_ before and after PE adsorption is stronger than that of H_2_O. This is inconsistent with the results of interaction energy analysis (Section 4.1). Because the difference between the interaction energies of O_2_ and H_2_O is relatively small, thus, effects of the interaction energies on the diffusion of both two molecules are not decisive. However, the size of O_2_ molecule is smaller than that of H_2_O molecule; therefore, O_2_ molecule is easier to diffuse in PE. The diffusion coefficients of O_2_ and H_2_O in adsorbed PE are all smaller than those in pure PE. In order to better understand the influence of flavor organic molecules on the diffusion of O_2_ and H_2_O in PE, the *FFV* of PE and the interaction energies of O_2_ or H_2_O with flavor organic molecules were further analyzed.

The *FFV* of PE cells, before and after flavor adsorption, was calculated with a probe radius of 1.0 Å and listed in Table 8. The interaction energies of D-limonene, myrcene, ethyl hexanoate, 2-nonanone and linalool with O_2_ or H_2_O were calculated by MD simulation and were listed in Table 9.

Table 8 shows that the *FFV* of PE cell decreased with the loading of O_2_, while increased with the loading of H_2_O. This is because the alkane chain has strong hydrophobicity, and the free volume becomes larger after loading H_2_O. When flavor molecules were diffused in PE, the *FFV* of PE cell decreased, which led to the decrease in the diffusion coefficients of O_2_ and H_2_O.

**Figure 9:**
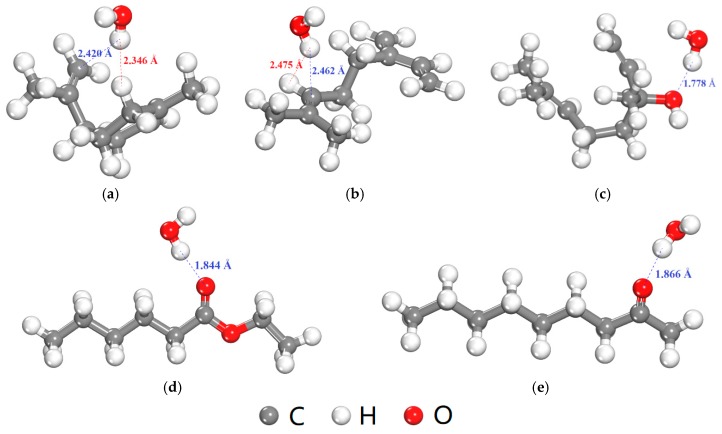
Equilibrium adsorption configurations of H_2_O molecule with five flavor organic molecules (the blue and red dash lines show attraction and repulsion interaction, respectively, determined by a distance criterion of 2.5 Å). (**a**) D-limonene; (**b**) Myrcene; (**c**) Linalool; (**d**) Ethyl hexanoate; (**e**) 2-nonanone.

In addition to the *FFV*, the interaction between O_2_ or H_2_O molecule and flavor organic molecules also affects the diffusion coefficients of O_2_ or H_2_O. Table 9 shows the interaction energies between O_2_ or H_2_O molecule and five flavor organic molecules. All the interaction energies were negative, which indicates the presence of the flavor organic molecules will hinder the diffusion of O_2_ and H_2_O molecules, and thus, lead to the decrease in the diffusion coefficients (Table 8). Besides, the interaction energies between H_2_O and five flavor organic molecules were stronger than those between O_2_ and five flavor organic molecules, especially for ethyl hexanoate, 2-nonanone and linalool. The equilibrium adsorption configurations of H_2_O molecule with five flavor organic molecules were shown in Figure 9. The H atom in H_2_O molecule is positively charged and expected to absorb near to the negatively charged C atoms in D-limonene and myrcene (Figure 9a,b). O atom of higher electronegativity is contained in the ethyl hexanoate, 2-nonanone and linalool molecules, which can form the hydrogen bond with the H atom in H_2_O (Figure 9c–e). Therefore, the interaction between H_2_O and ethyl hexanoate, 2-nonanone and linalool molecules are stronger than those of D-limonene and myrcene. Therefore, in a word, the presence of the flavor organic molecules results in greater hindrance to the diffusion of H_2_O compared with the case of O_2_, which can further explain the greater decrease in the diffusion coefficient of H_2_O in the presence of flavor organic molecules (Table 7).

## 5. Conclusions

The diffusion of D-limonene, myrcene, ethyl hexanoate, 2-nonanone and linalool in LDPE were investigated by two-sided immersion experiment, Brandsch prediction model and MD simulation. The diffusion of O_2_ and H_2_O in PE before and after the adsorption of the flavor substances were also studied. The following conclusions were drawn: (1) the immersion experiment showed the diffusion of five flavor molecules in LDPE well followed Fick’s second law; (2) MD simulation indicated the dual mode diffusion mechanism of the flavor molecules in LDPE; (3) the diffusion coefficients from MD simulation are close to those from the experimental determination, but are slightly larger than those values; (4) the presence of the flavor organic molecules hindered the diffusion of O_2_ and H_2_O, which can be well explained from *FFV* and interaction energy calculation results derived from MD simulation.

## Figures and Tables

**Table 1:** Composition of the amorphous cells constructed.

Model	Composition							
	PE160	D-LE	ME	EH	2-NO	LO	O_2_	H_2_O
PE + flavor	3	1	1	1	1	1	0	0
PE + O_2_	3	0	0	0	0	0	3	0
PE + H_2_O	3	0	0	0	0	0	0	10
PE + flavor + O_2_	3	1	1	1	1	1	3	0
PE + flavor + H_2_O	3	1	1	1	1	1	0	10

**Table 2:** Interaction energies between flavor, H_2_O, O_2_ molecules, and PE chain.

Molecules	*E*_int_ (kcal/mol) with PE Chain
D-limonene	−1.45
Myrcene	−1.98
Ethyl hexanoate	−2.00
2-nonanone	−1.88
Linalool	−2.96
O_2_	−0.83
H_2_O	−0.25

**Table 3:** Diffusion coefficients (*D*_pre_) calculated by Brandsch model.

Flavor Molecules	*M*_r_	*D*_pre_ (cm^2^/s)
D-limonene	136.24	1.91 × 10^−8^
Myrcene	136.23	1.91 × 10^−8^
Ethyl hexanoate	144.21	1.70 × 10^−8^
2-nonanone	142.24	1.75 × 10^−8^
Linalool	154.25	1.48 × 10^−8^

**Table 4:** Diffusion coefficients (*D*_exp_) fitted by experimental data.

Flavor Molecules	*D*_exp_ (cm^2^/s)	*R* ^2^
D-limonene	(6.69 ± 1.63) × 10^−11^	0.98
Myrcene	(5.72 ± 2.18) × 10^−11^	0.96
Ethyl hexanoate	(3.08 ± 1.14) × 10^−11^	0.99
2-nonanone	(3.50 ± 2.15) × 10^−11^	0.95
Linalool	(5.41 ± 2.54) × 10^−11^	0.97

**Table 5:** Comparison between experimental, prediction model and MD simulation results.

Molecules	*D*_pre_ (cm^2^/s)	*D*_sim_ (cm^2^/s)	*D*_exp_ (cm^2^/s)	*D*_pre_/*D*_exp_	*D*_sim_/*D*_exp_
D-limonene	1.91 × 10^−8^	(9.31 ± 0.24) × 10^−11^	(6.69 ± 1.63) × 10^−11^	286	1.39
Myrcene	1.91 × 10^−8^	(7.90 ± 0.13) × 10^−11^	(5.72 ± 2.18) × 10^−11^	334	1.38
Ethyl hexanoate	1.70 × 10^−8^	(6.90 ± 0.13) × 10^−11^	(3.08 ± 1.14) × 10^−11^	552	2.24
2-nonanone	1.75 × 10^−8^	(1.56 ± 0.02) × 10^−10^	(3.50 ± 2.15) × 10^−11^	500	4.46
Linalool	1.48 × 10^−8^	(1.72 ± 0.04) × 10^−10^	(5.41 ± 2.54) × 10^−11^	274	3.18

**Table 6:** *FFV* of PE cell with the different probe radius.

*R*_P_	1 Å	1.5 Å	2 Å	2.7 Å
*FFV* (%)	18.53	7.70	2.58	0.46

**Table 7:** Diffusion coefficients of O_2_ and H_2_O before and after adsorption of PE by flavor.

Molecules	*D*_before_ (cm^2^/s)	*D*_after_ (cm^2^/s)
O_2_	4.04 × 10^−10^	2.38 × 10^−10^
H_2_O	2.93 × 10^−10^	1.12 × 10^−10^

**Table 8:** *FFV* of PE cell before and after adsorption.

Form	PE	PE + O_2_	PE + H_2_O
*FFV* (%) before adsorption	18.49	17.92	19.75
*FFV* (%) after adsorption	17.18	16.42	17.68

**Table 9:** Interaction energies between O_2_ or H_2_O and flavor molecules.

Flavor Molecules	*E*_int_ (kcal/mol) with O_2_	*E*_int_ (kcal/mol) with H_2_O
D-limonene	−1.01	−2.42
Myrcene	−1.06	−1.85
Ethyl hexanoate	−0.72	−4.74
2-nonanone	−0.91	−5.52
Linalool	−0.60	−6.67
